# Social entrepreneurial intention among working adults: An emerging country context

**DOI:** 10.3389/fpsyg.2023.1123198

**Published:** 2023-02-13

**Authors:** Qing Yang, Abdullah Al Mamun, Gao Jingzu, Long Siyu, Muhammad Mehedi Masud

**Affiliations:** ^1^Graduate School of Business, Universiti Kebangsaan Malaysia, Bangi, Malaysia; ^2^Faculty of Business and Economics, University of Malaya, Kuala Lumpur, Malaysia

**Keywords:** value-belief-norm model, social entrepreneurship, entrepreneurial intention, working adults, social norms

## Abstract

Under the premise of the value-belief-norm (VBN) model, this study examined the influence of values, beliefs and norms on social entrepreneurial intention of working adults in China. The cross-sectional design was employed, and an online survey, which involved 1,075 working adults, was conducted. All data were analyzed using partial least squares-structural equation modeling (PLS-SEM). The obtained results showed the significant and positive influence of self-enhancement, openness to change, and self-transcendence on the sense of meaning and purpose. Moreover, the sense of meaning and purpose exhibited significant and positive influence on problem awareness, and problem awareness was found to have positive effect on outcome efficacy. Besides that, the sense of meaning and purpose, problem awareness, outcome efficacy, and injunctive social norms were found to exhibit significant and positive on personal norms. Finally, personal norms and injunctive social norms exhibited statistically significant and positive influence on social entrepreneurial intention. The results of effect size confirmed the considerable influence of personal norms and injunctive social norms on social entrepreneurial intention. Therefore, policy development aimed at promoting socioeconomic and environmental sustainability through social entrepreneurship should comprehensively consider the influence of personal norms and injunctive social norms. Increasing the sense of meaning and purpose of the working population, prompting an increased problem consequence and outcome self-efficacy, as well as instilling personal norms and injunctive social norms through various social and environmental incentives are recommended.

## Introduction

Social entrepreneurship is a rapidly growing area of academic research, practice, and policy development in the field of entrepreneurship (Hu et al., 2019). Social enterprises are defined as opportunities to catalyze social change and development with the aim of creating social value (Hockerts, 2017; Hu et al., 2019). The initial rise of social entrepreneurship globally was driven by issues of government failure, market failure, and excessive market competition, leading to an increase in demand for non-profit services in high welfare states and markets; thus, providing a viable environment for the creation and proliferation of non-profit organizations (Bacq and Janssen, 2011). As compared to commercial entrepreneurship, which is considered to be profit-oriented, social entrepreneurship is more focused on the current needs of society and aims to solve social problems (Hu et al., 2019). Therefore, exploring social entrepreneurial intention (SEI) of the masses can provide a more effective and comprehensive understanding of public perceptions of social entrepreneurship and needs of the current society, while helping to address the social problems caused by government failure, market failure, and excessive market competition (Hockerts, 2017; De Bernardi et al., 2021).

Poverty, environmental problems, poor sanitation, health deficiencies, and a number of other social issues are currently a global concern, and how to address these social issues is a concern for many countries (De Bernardi et al., 2021). Due to the nature of the work programes, alleviating poverty and solving social problems are the aims and objectives of social entrepreneurs (Diochon, 2013; Rawhouser et al., 2017). Prior studies demonstrated that the majority of social entrepreneurs aim to solve social problems through their business plans and ventures given their key role in solving social problems and providing aids, financial assistance, and necessities to the poor sections of the society (Cherrier et al., 2018). Social enterprises and social entrepreneurs not only contribute to the society but also strengthen the economic activities of the country and contribute economic impact on the overall economy of a country–for example, social enterprises introduce social innovation through their product and service innovation (Dwivedi and Weerawardena, 2018). Social entrepreneurship can be promoted as a form of entrepreneurship that can be profitable in terms of both economic and social returns, which in turn leads individuals to embark on the exploration and investigation of social activities. Therefore, it is essential to study and explore factors that lead to the formation of SEI.

Social enterprises have emerged as a new form of organization to serve social, economic, and environmental needs through profitable business operations (Wang et al., 2016). After nearly a decade of rapid economic development, social entrepreneurship has grown well in China and played a highly significant role in promoting the solutions of social issues, such as education, disability assistance, employment, poverty alleviation, and environmental protection (Warnecke, 2018). Social entrepreneurship integrates the attributes of social mission and corporate profitability and is considered a new concept to address the growing social and economic problems in developing countries like China (Crupi et al., 2021). It has become an important force to improve social welfare and promote stable social development. Controversy in SE-related research has centered on whether governments can introduce policies or intervene specifically to promote entrepreneurs to address pressing social needs, yet governments tend to be more active in promoting SE to generate social value and focus on unaddressed social needs, especially in emerging countries that are actively developing such as China, India, and Bangladesh (Crupi et al., 2021; Tu et al., 2021). Among them, the Chinese government has been very supportive of social entrepreneurship, which in turn has facilitated social entrepreneurial enterprises to continuously engage in social entrepreneurship-related behaviors in response to the social needs that continue to arise in the current society (Crupi et al., 2021). As social entrepreneurship becomes more popular and widespread in China, the dynamic market environment and accelerated iterative technological innovations have prompted organizations and employees to take into account social entrepreneurship, resulting in the identification of new opportunities to achieve sustained superior performance (Warnecke, 2018). Focusing on working adults in China, the current study explored their perceptions of social entrepreneurship and examined factors that influence SEI.

In order to fill the research gap in terms of working adults’ SEI, the main purpose of this study was to examine their intention and influencing factors of social entrepreneurship in the work process using the extended value-belief-norm (VBN) model. Previous studies have examined and explored entrepreneurs’ entrepreneurial intentions, as well as entrepreneurial motivations and influences (e.g., personal cognitive factors) by using TPB theory, which in turn predicts subsequent social entrepreneurial behavior and factors that influence social entrepreneurial intentions (Entrialgo and Iglesias 2016; Barton et al., 2018; Al-Jubari, 2019). These studies have successfully provided insight into the formation of social entrepreneurial intentions and how to propose strategies to motivate future social entrepreneurs. However, it is evident from previous studies that the use of the TPB model alone is not sufficient; these studies did not take into account the personal factors of entrepreneurs (e.g., values, perceptions of their beliefs), so this study introduces the VBN model to explore the influence of factors such as values on the social entrepreneurial intentions of working staff. In this study, the extended VBN model was used to examine the relationships of extended values, beliefs, and norms with SEI of working adults at multiple levels. In this proposed VBN model, self-enhancement (SFE), openness to change (OTC), self-transcendence (SET), sense of meaning and purpose (SMP), problem awareness (PRA), and outcome efficacy (OCE) were introduced and applied as extended value and belief factors to examine personal norms (PRN) in detail. Apart from PRN, injunctive social norms (ISN) were also incorporated into the model to evaluate the influence of different types of norms on SEI.

## Literature review

### Theoretical foundation

Considering the study of entrepreneurial intentions and behaviors, most researchers choose to use the Theory of Planned Behavior (TPB) and confirm that explanatory TPB models are much better at explaining conservation behaviors (Kaiser et al., 2005; Barton et al., 2018; Al-Jubari, 2019). However, intention-based models (e.g., TPB) do not fully explain the reasons why people engage in social entrepreneurship as a type of behavior, such as whether a person pursues entrepreneurial behavior out of personal choice (autonomous/intrinsic reasons) or out of obligation (controlled/extrinsic reasons; Al-Jubari, 2019). Therefore, many studies have proposed combining TPB with other theories or models to obtain a more comprehensive and in-depth view of a person’s behavioral intentions (Fayolle and Liñán, 2014; Al-Jubari, 2019). Among others, Fayolle and Liñán (2014) found that values explain the formation of intentional antecedents (e.g., attitudes and norms) and also indirectly influence entrepreneurial intentions, and that values can also play a role in the intention-action link. Therefore, in order to understand and explore in more detail the influence of intrinsic personal reasons such as values on the social entrepreneurial intentions of the study participants, this study introduces and applies the VBN theory, which in turn refines and deepens the research on social entrepreneurial intentions.

In most past studies, the VBN theory model has been commonly used to study individuals’ conservation intentions and conservation behaviors, such as environmental protection and conservation behavior (Ghazali et al., 2019), green consumption intentions and behaviors (Abutaleb et al., 2021), and pro-environmentally friendly apparel product purchasing behavior (Kim and Seock, 2019). In these studies, VBN theory explains the acceptability and expected influence of personal factors such as values on individuals’ intentions and behaviors such as pro-environmental categories, while predicting adjacent beliefs and other beliefs and norms downstream of the causal chain, suggesting that VBN theory can effectively explain the role that values play in promoting individuals’ intentions and behaviors (Kim and Seock, 2019; Abutaleb et al., 2021). Therefore, in this study, VBN theory is used as the main research theory to investigate the factors that influence the social entrepreneurial intentions of the Chinese workforce. The VBN model can be viewed as a causal chain of values, beliefs, norms, and behaviors. Values represent individuals’ perceptions of values (Stern et al., 1999; Ghazali et al., 2019). The emergence of the VBN theory illustrates the antecedents of social movement support and individual environmentalism, and the widespread use of the VBN theory has led to its prominence as a social psychological theory (Abutaleb et al., 2021).

As for the values component, this study considers SFE, OTC, and SET based on Schwartz’s fundamental values theory (Stern et al., 1999). Although previous studies have yielded a direct relationship between values and behavior, this relationship is stronger in the presence of other mediating variables, such as specific beliefs or PRN (Stern et al., 1999; Choi et al., 2015). Beliefs refer to a person’s thoughts about the natural environment and human behavior. Drawing on this, the present study considered SMP, PRA, and OCE. Individuals influenced by social norms are more likely to acquiesce to the opinions or advice of their significant others, such as family members, close friends, colleagues, and peers, which in turn influence their intentions and actions toward certain behaviors (Ghazali et al., 2019; Kim and Seock, 2019). Therefore, the present study incorporates ISN into the model and examines its effects on SEI.

### Development of hypotheses

#### Values: Self-enhancement

SFE is one of the egoistic values–there are two main types of values, namely power and achievement (Golob et al., 2018; Ünal et al., 2019). The motivational goal of power focuses on gaining control or dominance over people and resources, whereas the motivational goal of achievement focuses on achieving personal success through personal competence or external abilities (Schwartz, 1992). These values influence others’ attitudes, intentions, and behaviors in different ways. Furthermore, power is the antithesis of universalism in Schwartz’s (1992) value structure, representing the highest degree of SFE.

#### Values: Openness to change

OTC refers to stimulation and self-direction based on the motivation of independent thoughts and actions, which conflict with the motivation of fulfilling others’ expectations (Parks-Leduc et al., 2014; Canlas et al., 2022). The concept of OTC or readiness to change has been widely adopted in various studies. In a recent study, it was found that OTC can include creative behavior, open-mindedness, and willingness to try new things and take risks (Kautish and Sharma, 2019). OTC consists of two main dimensions of self-direction and stimulation, which involve intrinsic motivation for personal beliefs and behaviors (Kautish and Sharma, 2019; Tewari et al., 2022).

#### Values: Self-transcendence

SET is a socio-altruistic value orientation that is closely related to what has been defined as pro-social in another line of study (Golob et al., 2018). The former value orientation includes universalism and benevolence, and the motivational goal of universalism is to enhance the welfare of all people. Gärling (1999) found the equal inclination of pro-social and pro-ego individuals toward benevolence; however, pro-social individuals in this study were more inclined toward universalism, suggesting an important difference between different types of values. The study further reported the positive relationship of universalism with pro-environmental attitudes and behaviors, implying the influence of SET on one’s attitudes, intentions, and behaviors. Prior studies measured and studied SET and confirmed the importance of SET and the likelihood of individuals achieving various goals, as well as the intensity of motivation to work toward these goals (Zammitti et al., 2021).

#### Beliefs: Sense of meaning and purpose

A SMP in life refers to the perception and awareness of the world, the feeling and understanding of value, as well as the experience of self-belief (George and Park, 2016). The four potential motivational directions of values (Schwartz, 1992) and earlier related studies suggested a relationship between values and SMP. Prior studies divided “meaning” into four levels, each corresponding to different type of motivation and value (Makransky et al., 2019) and different perception of meaning. According to Schwartz’s (1992) theories, it can link the depth of sense of meaning and values together and divide the sense of meaning into four levels of depth, which include comfort, enhancement of personal potential, serving others in the immediate environment, and serving the universal good. By stratifying Schwartz’s 10 basic values and sense of meaning, Besika et al. (2022) linked these four levels of sense of meaning in depth to four motivational orientations, including SFE, OTC, and SET (Besika et al., 2022). According to Besika et al. (2022), SFE, OTC, and SET influence SMP in different levels of value domains. Thus, the relationships of SFE, OTC, SET, and SMP were hypothesized as follows:

*H1*: Self-enhancement positively influences the sense of meaning and purpose.

*H2*: Openness to change positively influences the sense of meaning and purpose.

*H3*: Self-transcendence positively influences the sense of meaning and purpose.

#### Beliefs: Problem awareness

PRA is defined as the degree to which an individual is aware of the adverse consequences of not acting pro-socially for others or for other things they value (Taso et al., 2020). PRA can motivate individuals to adopt new social behaviors and actions by activating PRN or perceived moral obligations (Kim and Hwang, 2020). Personal norms can be an internalized social norm or norm that is derived from higher-order values and perceived to be derived from general and environmental values (Schwartz, 1977).

#### Beliefs: Outcome efficacy

OCE is defined as identifying the need to act in order to alleviate what others or one-self value. Schwartz (1977) and Schwartz and Howard (1980) conceptualized OCE as the identification of specific actions that can alleviate the identified problem. Certain recent studies have argued that this definition is too limited for large-scale social problems because people may not be aware of possible actions and the value of those actions; therefore, OCE has been conceptualized as the degree of control one has over the problem (Steg and Nordlund, 2018). OCE is particularly important for large-scale problems that can only be solved when many people cooperate, such as reduction of harmful emission and charity donation. In such cases, perceived control over the outcomes heavily depends on the expectation that others engage in pro-social actions. Indeed, the existence of many social problems makes it necessary to examine the role of OCE (Steg and Nordlund, 2018; Joanes et al., 2020).

Nordlund and Garvill (2003) confirmed the influence of SET and environmental values on PRA. A relationship between SET and SMP was predicted to exist, and in turn, SMP and PRA were hypothesized to be positively correlated. De Groot and Steg (2009) confirmed the significant relationship between PRA and PRN and identified OCE as a mediator to influence the relationship between PRA and PRN. When PRA is high, individuals consider whether effective actions can be taken to reduce the problem (OCE). Furthermore, the proposed model theoretically confirms that an individual is less likely to feel responsible for pro-social behaviors or consider the validity of possible actions without knowing whether pro-social behaviors are present (De Groot and Steg, 2009). Therefore, the relationships of SMP, PRA, and OCE were hypothesized as follows:

*H4*: The sense of meaning and purpose positively influences problem awareness.

*H5*: Problem awareness positively influences outcome efficacy.

#### Norms: Personal norms

Social norms and PRN greatly influence human behaviors. PRN are values that individuals set according to their personal moral standards, which in turn influence their thoughts, actions, and behaviors (Shenaar-Golan and Walter, 2020). Based on the normative activation model, PRN refer to one’s sense of self-moral obligation to perform pro-social actions, suggesting that PRN are self-expectation and reflect their sense of responsibility to perform specific actions (Schwartz, 1977).

According to Jansson and Dorrepaal (2015), PRA at different levels is a pre-condition for the development of environmental attitudes and norms, which implies the relationship between PRA and PRN and that PRA can influence one’s intention and behavior through norms (Si et al., 2020). Nordlund and Garvill (2003) found a positive relationship between general and specific PRA and the positive influence of specific PRA on PRN. Meanwhile, Steg et al. (2016) identified OCE as a mediator of success in the attribution of responsibility in the VBN model involving consequence of awareness and PRN. The study also confirmed the relationship between OCE and PRN. Numerous prior studies explored the relationship between beliefs and norms and confirmed an inextricable relationship between factors related to beliefs and norms, which in turn corroborated that different belief factors have different degrees of influence on PRN (Gkargkavouzi et al., 2019). Thus, the following hypothesis was proposed:

*H6*: The sense of meaning and purpose positively influences personal norms.

*H7*: Problem awareness positively influences personal norms.

*H8*: Outcome efficacy positively influences personal norms.

#### Norms: Injunctive social norms

ISN indicate perceptions about normatively appropriate behavior in a specific context. These norms reflect what kind of behavior is approved or disapproved by the community, which motivate actions through the anticipation of social rewards or punishment (Heinicke et al., 2022). ISN indicate the perceptions of others’ attitudes and behaviors, and this factor is critical to the formation of social norm perceptions (Cialdini, 2003; Cho et al., 2015). The importance of ISN in the field of environmental behavior has been consistently demonstrated. The role of ISN in motivating and altering behaviors in a range of contexts has been highlighted in numerous studies (Blanton et al., 2008; Cho et al., 2015; Heinicke et al., 2022). Accordingly, it can be reasonably predicted that ISN influence one’s intention and behavior.

#### Injunctive social norms and personal norms

Schwartz (1977) defined PRN as self-based standards of a certain behavior, which are derived from personal values and beliefs and enforced through expected SFE or self-depreciation. Several prior studies demonstrated the moderating role of PRN in the relationship between ISN and behaviors (Bertoldo and Castro, 2016; Bonan et al., 2020; Niemiec et al., 2020; De Groot et al., 2021). According to the existing social psychological theories, a few prior studies confirmed that social norms are processed differently from PRN. Their findings suggest that social norms can influence behaviors at a more subconscious level through the influence of emotional beliefs (Oh et al., 2021), and social norms can be internalized into PRN (Thøgersen, 2006). PRN can consciously motivate one’s intention and behavior due to the individual tendency to be more closely associated with cognitive beliefs (De Groot et al., 2021). Many prior studies considered social norms as antecedents of PRN and reported the influence of social norms on PRN by examining the relationship between these two constructs (Kim and Seock, 2019; Morren and Grinstein, 2021). Based on the findings of previous studies, the current study predicted a potential relationship between ISN and PRN:

*H9*: Injunctive social norms positively influence personal norms.

##### Social entrepreneurial intention

Social entrepreneurship has become an increasingly common way to meet social and economic needs (Rawhouser et al., 2017). Most of the prior studies on intentions and behaviors applied the theory of planned behavior because this theory confirms that intention predicts behavior (Ajzen and Fishbein, 1970). Entrepreneurship can be seen as an effective tool for creating economic value (Nicholls, 2010). Social entrepreneurial intentions are often considered as a means to deal with various social issues (Tiwari et al., 2017) and it helps to overcome inequalities and bridge gaps in different fields (Nicholls, 2010). Additionally, the importance of intention is that it is one of the key factors that predict planned behavior (Krueger and Brazeal, 1994). SEI refers to a state of mind, particularly the desire and determination of an individual to pursue a social mission, which guides and directs entrepreneurial actions to create social enterprises (Bacq and Alt, 2018). Hockerts (2017) confirmed that interventions that expose individuals directly to social issues may cause an increase in SEI, such as volunteering programes, and that there may be differences between the effects of both perceived ISN and PRN in peer groups on SEI. Unlike other prior studies on SEI, the current study used the VBN model to examine the SEI of working adults in China in order to understand their needs and perceptions in relation to SEI and to examine the relationships of values, beliefs, and norms with SEI.

#### Social entrepreneurial intention and norms

Prior studies that focused on the relationships of norms, intentions, and behaviors confirmed the varying effects of different norms on individuals’ intentions and behaviors (Niemiec et al., 2020; Heinicke et al., 2022). In the VBN model, the positive relationship between PRN and behavioral intention has been confirmed in many past studies of pro-environmental behavioral intentions, such as intention to use green hotels (Bashir et al., 2019) and intention to buy environmentally friendly products (Kim and Seock, 2019). Therefore, it can be hypothesized that there is a significant relationship between PRN and behavioral intention, i.e., PRN can significantly and positively influence SEI. Social norms in the form of ISN were confirmed in Wong’s (2019) study, can significantly influence individuals’ behavioral intentions, implying that there may be a potential relationship between ISN and behavioral intentions in the category of social entrepreneurship. Bazan et al. (2020) confirmed that ISN is usually caused by the influence of others, which in turn affects individuals’ behavioral intentions and subsequent behaviors. That is, both the external environment and external influences can cause changes in an individual’s ISN, which in turn affects the individual’s behavioral intentions. Considering that, ISN may have the same effect on the pro-social type of behavior, such as social entrepreneurship. Consequently, the relationship between norms and SEI was hypothesized as follows:

*H10*: Personal norms positively influence social entrepreneurial intention.

*H11*: Injunctive social norms positively influence social entrepreneurial intention.

**Figure 1:**
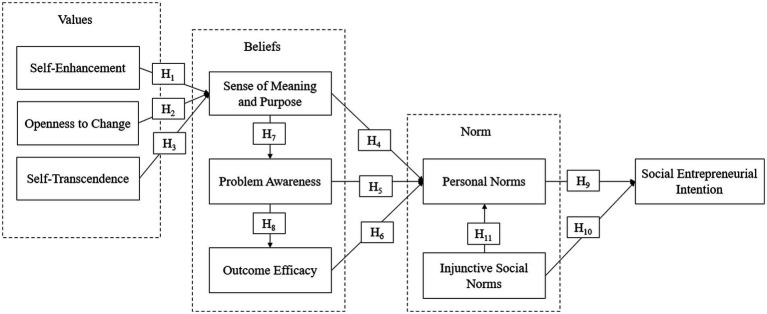
Research framework.

Based on the review of related literature, the proposed research framework in this study was divided into four main sections. As shown in Figure 1, a total of 11 hypotheses were developed for testing.

## Methodology

### Sample selection and data collection

Adopting the cross-sectional design, this quantitative study targeted working adults in China. An online questionnaire survey was conducted following the constraints of the COVID-19 pandemic. All questionnaire items were adapted from prior studies and translated into Chinese in collaboration with a professional English-speaking institution to better suit the target population of this study. A pre-test was conducted to ensure the reliability and validity of the Chinese version of the questionnaire survey prior to the actual data collection. Following that, an online questionnaire survey was uploaded using the WJX website^1^.

This study employed the non-probability sampling strategy, specifically a combination of snowball and judgmental sampling strategies. There were no specific employment requirements in terms of years, job type, or gender for the selection of respondents for this study. The only requirement set in this study was that the respondents must be full-time employees of age 18 years and above. The consistency of the collected data were examined; any cases of respondents replying the same responses to all questions were remove. As a result, this study successfully gathered a total of 1,075 valid questionnaire sets.

The questionnaire set consists of three main sections: (1) a description of the questionnaire survey; (2) a set of background questions; (3) a set of scale questions. Before the respondents began to answer the questions in the questionnaire survey, they were informed of the purpose of the questionnaire survey and required to sign an informed consent in the first section of the questionnaire survey. All data were kept anonymous and confidential, and the respondents may opt out of the questionnaire survey at any point during their participation. Furthermore, the definition and explanation of social entrepreneurship are included in the description to assist the respondents in comprehending the content of the questionnaire survey. The second section focuses on the demographic characteristics of the respondents, such as their age, gender, education level, average monthly income, and location of residence. In the third section of the questionnaire survey, the respondents were required to provide their responses that best reflect their values, beliefs, norms, and intention to engage in social entrepreneurship according to a seven-point Likert scale.

### Survey instrument

The measurement items in this study were adapted from previous studies. Furthermore, two different sets of seven-point Likert scale, with the endpoints of “not very important” (1) and “very important” (7) and the endpoints of “strongly disagree” (1) and “strongly agree” (7), were employed. Several measurement items were adjusted and translated into Chinese. The initial draft of the questionnaire set was pre-tested, which involved the participation of 22 respondents, to determine the reliability and validity of the instrument after adaptation and translation. The following prior studies were selected for the current study to obtain specific measurement items: SFE (Lindeman and Verkasalo, 2005); OTC (Lindeman and Verkasalo, 2005); SET (Lindeman and Verkasalo, 2005); SMP (Dong et al., 2017); PRA (Ünal et al., 2017); OCE (Ünal et al., 2017); ISN (Doran and Larsen, 2015); PRN (Ünal et al., 2017); SEI (Ruiz-Rosa et al., 2020).

### Common method bias

The questionnaire survey was doubly specific because the study aimed to comprehend the intention to engage in social entrepreneurship among working adults. Firstly, when the issue of social entrepreneurship is left unexplained, it may lead one to choose the more “socially responsible” and “correct” options. Secondly, when it comes to surveying SEI among employed individuals, they are more like to be concerned about their anonymity and confidentiality of their responses. Bias-related factors that may affect the motivation of respondents to respond must be considered and avoided to the greatest extent possible. In this study, prevention and diagnosis of common method variance were performed before and after the questionnaire survey. Therefore, this study employed both procedural and statistical remedial measures to prevent this issue.

Procedurally, this study emphasized anonymity and confidentiality. All responses were treated equally and used exclusively for the purpose of this study. Besides that, an online questionnaire survey, rather than the conventional paper-based questionnaire survey, was adopted in this study to minimize bias caused by the respondents’ sensitivity to the research topic. This further emphasized confidentiality. Addin to that, all respondents were informed of the consent form and that none of the questions in the questionnaire survey have correct or incorrect answers. The respondents were informed that they were only required to answer the questions according to their true thoughts. Finally, this study cooperated with professional translation agencies, and conduct pre-test before the actual data collection.

Statistically, this study performed the Harman’s single-factor test, which revealed that the highest item percentage of variance (explanation rate) was 38.437% (less than 40; Fuller et al., 2016). At the same time, this study conducted a full collinearity test, as suggested by Kock (2015). As shown in Table 1, the recorded values of variance inflation factor (VIF) ranged from 1.511 to 2.175, which did not exceed the suggested threshold value of 3.3 (Kock, 2015). Overall, these results indicated the absence of common method bias in this study.

**Table 1:** Full collinearity test.

	SEF	OTC	SET	SMP	PRA	OCE	ISN	PSN	SEI
VIF	1.622	1.511	1.820	2.147	1.800	1.511	2.104	2.175	2.044

SFE, Self-Enhancement; OTC, Openness to Change; SET, Self-Transcendence; SMP, Sense of Meaning and Purpose; PRA, Problem Awareness; OCE, Outcome Efficacy; ISN, Injunctive Social Norms; PSN, Personal Norms; SEI, Social Entrepreneurial Intention. Author’s data analysis.

### Multivariate normality

Web Power online statistical tool^2^ was used for this study’s multivariate normality test prior to conducting PLS-SEM. The calculated *p*-values, as well as multivariate skewness and multivariate kurtosis were below 0.01 (below the recommended threshold value of 0.05), which indicated non-normality issue in this study.

### Data analysis methods

The study used software such as SPSS and SmartPLS for statistical analysis. The common methods bias tests and descriptive analyzes were carried out through SPSS software. The research model was evaluated and hypothesis tested through PLS-SEM. Compared to other research methods, the PLS-SEM method is suitable for optimizing the interpretation of the endogenous structure and variance of the predictive model and imposes fewer constraints on the multivariate normal distribution. Following a multivariate normality test (*p* < 0.05), the use of PLS-SEM for exploratory studies is the appropriate technique for this study. Modeling using PLS-SEM was divided into two main steps, the first of which was to evaluate the measurement model. After determining the reliability and validity of the measurement model, a structural model evaluation was conducted.

The study examined the linear correlation between all constructs (using the Fornell-Larcker Criterion and VIF values) and assessed the reliability and validity of the items in terms of convergent validity, internal consistency, and discriminant validity using the average variance extracted (AVE), Cronbach’s alpha, composite reliability, and Dijkstra-Henseler’s rho. Following the successful assessment of the model’s reliability and validity, the study further ran bootstrapping to evaluate the structural model, reporting the relationship between exogenous and endogenous constructs and testing the previously proposed hypotheses. In addition, compared to covariance based structural equation modeling (CB-SEM), PLS-SEM does not establish a global goodness-of-fit measure, which is particularly limiting when used for theoretical tests (Hair et al., 2022). The possibility of other endogeneities between exogenous and endogenous constructs is explored in detail.

## Results

### Demographic characteristics of respondents

Table 2 gives details of the demographic characteristics of the respondents. As for the age category, close to 78% of the respondents were between the ages of 20 and 60, with a concentration in the 31–40, 41–50 and 51–60 age groups, in line with the need to orient the data to the working population. The education level shows that 88.3% of the respondents have a secondary school certificate or above, indicating that they have the ability to think logically and read well enough to understand all the survey questions involved in the study and the right to informed consent. As for the average monthly income, nearly 75% of the respondents had a monthly income between CNY5,000 and CNY10,000, which equates to approximately USD750 to USD1,500. Financially, except for Shandong (18.0%; *N* = 194), the distribution of the respondents in this study was relatively even.

**Table 2:** Profile of the respondents.

	*N*	%		*N*	%
Gender			Average monthly income		
Male	444	41.3	Below CNY 2500	197	18.3
Female	631	58.7	CNY 2501–5,000	283	26.3
Total	1,075	100.0	CNY 5001–7,500	246	22.9
			CNY 7501–10,000	163	15.2
Age			CNY 10,001-12,500	106	9.9
20–30 years	55	5.1	Above CNY 12,501	80	7.4
31–40 years	225	20.9	Total	1,075	100.0
41–50 years	351	32.7			
51–60 years	208	19.3	Location		
Above 60 years	236	22.0	Beijing	82	7.6
Total	1,075	100.0	Shanghai	82	7.6
			Guangdong	69	6.4
Education			Guangxi	70	6.5
Secondary school certificate	126	11.7	Zhejiang	90	8.4
Diploma/Technical school certificate	258	24.0	Shandong	194	18.0
Bachelor degree or equivalent	501	46.6	Hunan	68	6.3
Master degree	138	12.8	Jiangsu	68	6.3
Doctoral degree	52	4.8	Others	352	32.7
Total	1,075	100.0	Total	1,075	100.0

### Measurement model (outer model)

Firstly, the multicollinearity between the models must be ruled out before the model structure can be evaluated. Thus, VIF was shown separately in this study, and the results are presented in Table 3. As the recorded values of VIF ranged from 1.000 to 1.913, which were significantly lower than the recommended threshold value of 5 (Hair et al., 2021), therefore indicate no significant multicollinearity issue between all constructs.

**Table 3:** Multicollinearity test.

Variables	VIF SMP	VIF PRA	VIF OCE	VIF PSN	VIF SEI
SFE	1.458	−	−	−	−
OTC	1.439	−	−	−	−
SET	1.454	−	−	−	−
SMP	−	1.000	−	1.844	−
PRA	−	−	1.000	1.799	−
OCE	−	−	−	1.913	−
PSN	−	−	−	−	1.555
ISN	−	−	−	1.733	1.555
SEI	−	−	−	−	−

SFE, Self-enhancement; OTC, Openness to change; SET, Self-transcendence; SMP, Sense of meaning and purpose; PRA, Problem awareness; OCE, Outcome efficacy; ISN, Injunctive social norms; PSN, Personal norms; SEI, Social entrepreneurial intention. Author’s data analysis.

Convergent validity, internal consistency, and discriminant validity were evaluated. For convergent validity, the recorded values of AVE (Table 4) ranged between 0.547 and 0.741, which exceeded the recommended threshold of 0.5, Concurrently, 50 items were retained in this study. Finings presented in Supplementary File 2 showed that the loading values of all items exceeded the cross-loadings, and all loading values exceeded 0.7, thus confirming the existence of convergent validity (Avkiran and Ringle, 2018). For internal consistency, Cronbach’s alpha, composite reliability, and Dijkstra-Henseler’s rho for all items in this study exceeded the threshold value of 0.7; thus, confirming the internal consistency of all items.

**Table 4:** Reliability analysis.

Variables	Items	Cronbach’s alpha	Dijkstra-Henseler’s rho	Composite reliability	AVE
SFE	5	0.869	0.877	0.905	0.655
OTC	6	0.840	0.867	0.877	0.547
SET	8	0.934	0.934	0.945	0.683
SMP	6	0.890	0.891	0.916	0.645
PRA	5	0.895	0.896	0.922	0.704
OCE	5	0.898	0.898	0.925	0.710
PSN	5	0.896	0.896	0.923	0.706
ISN	5	0.912	0.913	0.935	0.741
SEI	5	0.901	0.904	0.926	0.716

SFE, Self-enhancement; OTC, Openness to change; SET, Self-transcendence; SMP, Sense of meaning and purpose; PRA, Problem awareness; OCE, Outcome efficacy; ISN, Injunctive social norms; PSN, Personal norms; SEI, Social entrepreneurial intention. Author’s data analysis.

Finally, this study used the Fornell-Larcker criterion and the heterotrait-monotrait ratio (HTMT0.85) to evaluate the discriminant validity. The results of the Fornell-Larcker criterion are presented in Supplementary File 2. The square root of the diagonal values (the square root of AVE of the latent variables) were higher than that of the latent variables and other items. Meanwhile, referring to Supplementary File 2, the values of HTMT ranged from 0.161 to 0.750, which did not exceed the recommended threshold value of 0.85, therefore showed adequate discriminant validity (Avkiran and Ringle, 2018). The above results showed the reliability and validity of the measurement model in this study (Dijkstra and Henseler, 2015).

### Structural model (inner model)

This study used path coefficients, *R*^2^, *f*^2^, and *Q*^2^ to evaluate the structural model and examined the correlations between latent variables. A path coefficient analysis was performed under a one-tailed test through the bootstrapping procedure. Overall, the obtained results revealed the significant and positive influence of SFE, OTC, and SET on SET and the significant and positive influence of SET, PRA, and OCE on PRN. Besides that, the influence of SET on PRA was found statistically significant and positive. The relationship between PRA and OCE and the relationships of PRN and ISN with SEI were also revealed to be statistically significant and positive.

### Hypothesis testing

Based on the results in Table 5, the coefficient of determination (*R*^2^) of the endogenous constructs in this study exceeded the lowest threshold value of 0.25. The study’s model explained 44.8% of total variance in SMP, 49.8% of total variance in PSN, 28.6% of total variance in PRA, 35.3% of total variance in OCE, and 48.6% of total variance in SEI. Overall, the above results indicated that the explanation of exogenous structure to endogenous structure in the proposed model was moderate.

**Table 5:** Path coefficients.

Hypothesis		Beta	Confidence interval	*t*	*p*	*R* ^2^	*f* ^2^	*Q* ^2^	Decision
**Determinants of SMP**									
H1	SFE - > SMP	0.192	(0.128, 0.248)	5.172	0.000		0.046		Supported
H2	OTC - > SMP	0.188	(0.136, 0.239)	6.051	0.000	0.448	0.044	0.285	Supported
H3	SET - > SMP	0.429	(0.373, 0.485)	12.429	0.000		0.230		Supported
**Determinants of PRA**									
H4	SMP - > PRA	0.535	(0.485, 0.579)	18.601	0.000	0.286	0.402	0.200	Supported
**Determinants of OCE**									
H5	PRA - > OCE	0.594	(0.549, 0.634)	22.543	0.000	0.353	0.545	0.248	Supported
**Determinants of PSN**									
H6	SMP - > PSN	0.228	(0.167, 0.289)	6.103	0.000		0.056		Supported
H7	PRA - > PSN	0.131	(0.061, 0.192)	3.317	0.000	0.498	0.019	0.348	Supported
H8	OCE - > PSN	0.218	(0.151, 0.281)	5.550	0.000		0.049		Supported
H9	ISN - > PSN	0.280	(0.216, 0.349)	7.052	0.000		0.090		Supported
**Determinants of SEI**									
H10	PSN - > SEI	0.396	(0.334, 0.450)	11.433	0.000	0.486	0.184	0.343	Supported
H11	ISN - > SEI	0.384	(0.328, 0.438)	11.604	0.000		0.196		Supported

SFE, Self-enhancement; OTC, Openness to change; SET, Self-transcendence; SMP, Sense of meaning and purpose; PRA, Problem awareness; OCE, Outcome efficacy; ISN, Injunctive social norms; PSN, Personal norms; SEI, Social entrepreneurial intention. Author’s data analysis.

The study used predictive relevance (*Q*^2^) to test the predictive ability of exogenous variables in the model to endogenous variables. The results showed that the values of *Q*^2^ for SMP (*Q*^2^ of 0.285), PSN (*Q*^2^ of 0.348), PRA (*Q*^2^ of 0.200), OCE (*Q*^2^ of 0.248), and SEI (*Q*^2^ of 0.343) exceeded zero and were rather high, suggesting the satisfactory and considerable predictive power of the measurement model (Sarstedt et al., 2017).

This study assessed effect size (*f^2^*) as a measure of the importance of exogenous structure in explaining endogenous structure. A widely used explanation is that, according to the criteria proposed by Cohen (2013), *f*^2^ that exceed 0.02, 0.15, and 0.35 imply small effect, medium effect, and large effect, respectively. SET (*f*^2^ of 0.046) to SMP, OTC (*f*^2^ of 0.044) to SMP, SMP (*f*^2^ of 0.056) to PSN, OCE (*f*^2^ of 0.049) to PSN, ISN (*f*^2^ of 0.090) to PSN had small effect size, whereas SET (*f*^2^ of 0.230) to SMP, PSN (*f*^2^ of 0.184) to SEI, ISN (*f*^2^ of 0.196) to SEI had medium effect size. Meanwhile, SMP (*f*^2^ of 0.402) to PRA and PRA (*f*^2^ of 0.545) to OCE had large effect size.

The results further showed the significant and positive influence of SFE [*β* = 0.192, *p* < 0.001, CI = (0.128, 0.248)], OTC [*β* = 0.188, *p* < 0.001, CI = (0.136, 0.239)], and SET [*β* = 0.429, *p* < 0.001, CI = (0.373, 0.485)] on SMP. In other words, H1, H2, and H3 were supported. The results also showed the significant and positive influence of SMP [*β* = 0.228, *p* < 0.001, CI = (0.167, 0.289)], PRA [*β* = 0.131, *p* < 0.001, CI = (0.061, 0.192)], and OCE [*β* = 0.218, *p* < 0.001, CI = (0.151, 0.281)] on PSN. Thus, H6, H7, and H8 were supported. At the same time, there was a significant and positive relationship between SMP [*β* = 0.535, *p* < 0.001, CI = (0.485, 0.579)] and PRA, which supported H4. Besides that, there was a significant and positive relationship between PRA [*β* = 0.594, *p* < 0.001, CI = (0.549, 0.634)] and OCE. Thus, H5 was supported. Adding to that, the results revealed the significant and positive influence of ISN [*β* = 0.208, *p* < 0.001, CI = (0.216, 0.349)] on PSN. In other words, H9 was supported. Finally, PSN [*β* = 0.396, *p* < 0.001, CI = (0.334, 0.450)] and ISN [*β* = 0.384, *p* < 0.001, CI = (0.328, 0.438)] exhibited significant and positive influence on SEI; thus, supporting H10 and H11. The recorded t-values for all hypotheses exceeded 3.090 (one-tail; 0.001), and the recorded minimum and maximum values of CI did not include zero. These results confirmed that all hypotheses were supported.

Considering that PLS-SEM is based on regressivity analysis, the discussion of the issue of endogeneity is an integral part of regression analysis (Hult et al., 2018). In this study, Gaussian copulas were introduced to identify possible endogeneity and the results are reported in Table 6. The data show no other emergence of endogeneity in the relationship between exogenous and endogenous constructions in all hypotheses except H2 and H3. Openness to change (*β* = −0.168, *p* = 0.037) has an unknown endogeneity in the relationship with sense of meaning and purpose This endogeneity weakened the effect of openness to change on sense of meaning and purpose. Meanwhile, the presence of an unknown endogeneity in the relationship between self-transcendence (*β* = 0.126, *p* = 0.004) and sense of meaning and purpose very weakly enhanced the effect of openness to change on sense of meaning and purpose. Purpose. Endogeneity can occur for a variety of reasons, primarily measurement error, common method bias, and unobserved heterogeneity (Hult et al., 2018). Previous analyzes of model evaluation have demonstrated the absence of common method bias in this study, and it is likely that there are other variables in this study that have an impact on sense of meaning and purpose that are being overlooked. However, when discussing the endogeneity between the remaining structural pathways and the predictive power of the model, it is easy to see that the presence of this endogeneity does not affect the other structural pathways, and in this case, there is no need to control for endogeneity, as an overemphasis on controlling for endogeneity would reduce the predictive power of the model (Ebbes et al., 2005; Hult et al., 2018).

**Table 6:** Gaussian Copula tests.

	Original sample	Sample mean	Standard deviation	t statistics	*p* values
GC (SEE) - > SMP	−0.106	−0.109	0.058	1.839	0.066
GC (OTC) - > SMP	−0.168	−0.165	0.081	2.081	0.037
GC (SFT) - > SMP	0.126	0.125	0.044	2.862	0.004
GC (SMP) - > PRA	0.000	−0.005	0.061	0.002	0.998
GC (SMP) - > PSN	−0.031	−0.029	0.064	0.486	0.627
GC (PRA) - > OCF	0.090	0.090	0.057	1.576	0.115
GC (PRA) - > PSN	0.111	0.111	0.061	1.806	0.071
GC (ISN) - > PSN	0.060	0.061	0.077	0.781	0.435
GC (ISN) - > SEI	0.049	0.047	0.062	0.787	0.431
GC (PSN) - > SEI	−0.021	−0.018	0.063	0.328	0.743
GC (OCF) - > PSN	0.024	0.019	0.058	0.413	0.680

SFE, Self-enhancement; OTC, Openness to change; SET, Self-transcendence; SMP, Sense of meaning and purpose; PRA, Problem awareness; OCE, Outcome efficacy; ISN, Injunctive social norms; PSN, Personal norms; SEI, Social entrepreneurial intention. Author’s data analysis.

## Discussion

This study used the VBN model to examine the relationships of values, beliefs, norms, and SEI. All hypothesized relationships in this study were found statistically significant and positive. Combined with the findings of earlier studies, a detailed interpretation of these results is discussed as follows.

Firstly, SFE was found to have significant and positive influence on SMP. Ghazali et al. (2019) and Besika et al. (2022) reported similar results, which established the crucial role of SFE in social entrepreneurship. It is more straightforward for individuals who value self-improvement to develop a sense of belief in achieving meaningful goals. At the same time, OTC in this study was found to exhibit substantial and positive impact on SMP, which was found to be in line with other previous studies on green consumption orientation and pro-environmental behavioral intention (Ghazali et al., 2019; Tewari et al., 2022). Finally, the relationship between SET and SMP was also found significant and positive in this study, which supported the findings of George and Park (2016) and Besika et al. (2022) on the direction of life meaning and satisfaction. Individuals who constantly have high SET tend to pursue higher goals, and the realization of life meaning and goals can be difficult for most individuals to achieve. The current study’s results showed that individuals with strong SFE, OTC, and SET are more likely to support social entrepreneurship from the SET perspectives.

Based on the obtained results of this study, SMP, PRA, and OCE were found to exhibit significant and positive influence on PSN. De Groot and Steg’s (2009) study on pro-social behavioral intention and De Groot et al. (2021) study on pro-environmental behavior reported similar results. Besides that, these results indicated that SET, PRA, and OCE directly affected PRN. In other words, values at different individual levels serve as motivation for individuals to generate pro-environmental PRN, which help individuals to adopt socially beneficial behaviors. At the same time, this study found that SMP also exhibited significant and positive influence on PRA, which was similarly reported by Nordlund and Garvill (2003). When individuals have a sense of purpose and meaning that is “higher” than the individual itself, such belief activates their pro-social PRN, resulting in the formation of pro-social behavior. The influence of PRA on OCE in this study was also found statistically significant and positive. De Groot and Steg (2009) focused on pro-social behavioral intention and reported similar results. Based on these findings, it is evident that individuals should be more encouraged to control themselves from the perspective of beliefs in order to understand and adopt pro-social behavior, to focus on the promotion of such behavior, while motivating themselves, and to realize the importance and necessity of pro-social behavior, resulting in the adoption of pro-social behavior.

Additionally, other notable findings of this study involved the driving influence of ISN for PSN and SEI, which not only emphasize the findings of previous studies (Bonan et al., 2020; Niemiec et al., 2020; De Groot et al., 2021; Oh et al., 2021) and also give directions for future practical applications. Mass media tends to make deviations from social norms and can evoke universal social condemnation. It places violators under strong social pressure; thus, serving to enforce compliance with social norms. For individuals to survive and thrive in groups, they must abide by the rules of interactions established by social norms. In the future, pro-social behaviors generated by individuals can be considered through the promotion of social norms.

Finally, this study found that boosting PRN and ISN enhanced SEI. Both PHS and ISN demonstrated favorable and substantial influence on individuals’ acceptance or support of SEI, as early as in terms of pro-environmental behaviors (Steg et al., 2016) and pro-social behaviors (De Groot and Steg, 2009; De Groot et al., 2021). Prior studies on green consumption (Choi et al., 2015) corroborated the above findings. PSN in this study had a positive role in promoting SEI. In a social group, under the influence of social rules and norms, individuals with strong awareness of PRN are more likely to demonstrate SEI given their higher tendency to follow social trends and be influenced by the encouragement of their significant others, resulting in the adoption of pro-social behaviors. At the same time, ISN in this study positively influenced SEI, suggesting its active role in promoting social entrepreneurship. Rimal and Yilma (2021) presented similar findings. Furthermore, earlier studies mostly used ISN to study group behavior and mainly targeted criminals and students (Cho et al., 2015; Heinicke et al., 2022).

## Implications

### Theoretical implications

Using the extended VBN model, this study contributed significant theoretical implications on the existing knowledge on SEI, particularly in China. Studies have demonstrated the influence of values, beliefs, and norms on pro-environmental and pro-social behavioral intentions and behaviors across cultures and countries. Focusing on the field of social entrepreneurship, this study adopted the VBN model to examine the relationships of values, beliefs, and norms, as well as the influence of norms on employees’ SEI. Based on the obtained findings, this study demonstrated the influence of various constructs in the extended VBN model on incumbent employees’ SEI.

For the initial VBN model, this study used SFE, OTC, and SET as partial extension factors of values to examine the influence of these values on beliefs. In prior studies on the VBN model, values were mainly divided into altruistic values, biosphere values, and egoistic values. In the current study, altruistic values and egoistic values were not studied directly. However, SET was selected to represent altruistic values, and SFE was selected to represent egoistic values. This made the selection of value-related factors in this study more in line with the research background of social entrepreneurship. Based on the results of this study, it can be concluded that SET, OTC, and SFE, as part of values, effectively positively influence beliefs, which in turn influence PRN and SEI.

When the belief part of the VBN model was extended, this study selected SMP, PRA, and OCE to assess their influence on PRN, and the interrelationships of these three constructs. The findings of this study were found to be largely consistent with those reported by De Groot and Steg (2009), which showed the influence of PRA and OCE associated with specific behaviors on various types of PRN, as well as pro-social and pro-environmental behavioral intentions. The results of this study demonstrated the significant relevance of the VBN model in explaining pro-social and pro-environmental behavioral intentions and behaviors. The model can also be applied to studies on pro-social behavioral intentions and behaviors, such as social entrepreneurship.

This study examined two types of norms, namely PRN and social norms, to assess the influence of norms on SEI. This study selected ISN to represent social norms. Studies have noted the central role of PRN as a moderating variable in the causal chain of the effects of values, PRA, and OCE on pro-social behavioral intention. SMP of value orientation is important to establish or enhance PRA and OCE of pro-social behaviors (e.g., social entrepreneurship) and the basis for PRN. However, previous studies rarely discussed ISN within the context of the VBN normative model. In this study, the original VBN model was extended with the intervention of ISN. This study added theoretical basis for future research to extend the VBN model.

### Practical implications

With the increase of social needs, social entrepreneurship can effectively alleviate and resolve social problems, such as education, disability assistance, employment, poverty alleviation, and environmental conservation. As a result, an increasing number of studies have shifted their attention to the field of social entrepreneurship. This study confirmed the influence of normative factors, such as PRN and ISN in the VBN model, on SEI. These findings reaffirmed the potential validity of many measures with the potential of influencing SEI. This study empirically proved the influence of personal values, beliefs, and norms on SEI. Personal values and norms can be strategically used to assist and inspire more individuals in China and other Asian countries to engage in social entrepreneurship.

Secondly, prior studies confirmed that intentions and motivations for pro-social behaviors may decline over time, implying the need to make strategic efforts to strengthen and maintain intentions and motivations for pro-social behaviors in order to meet social needs (Bolino and Grant, 2016; Hafenbrack et al., 2020). This study demonstrated that these efforts should strategically make use of the influence of personal values, beliefs, and norms to reinforce and maintain individuals’ intention to engage in pro-social behaviors like social entrepreneurship. This can subsequently promote the development of social entrepreneurship, create more employment opportunities, and contribute to the economic growth and social stability of a country, particularly China.

Additionally, this study provided practical insights for businesses that engage in social entrepreneurship and for the general population that intends to engage in social entrepreneurship. Social entrepreneurship enterprises can guide and educate their employees in terms of their beliefs and standards, raise their awareness and understanding of social entrepreneurship, and empower them to improve their personal performance and advancement through intrapreneurship and social entrepreneurship. These measures offer more impetus to China’s development and social stability.

## Limitations and recommendations for future research

This study encountered several limitations in targeting the SEI of working adults in China. Firstly, only a few prior studies in the field of entrepreneurship applied the VBN model to explore social entrepreneurship. Therefore, the findings on the relationships of the components of the VBN model with SEI have remained scarce, resulting in the lack of reference for the current study. Furthermore, the review of literature revealed more gaps on the relationships of values and beliefs with normative intention within the context of social entrepreneurship. This implies the underdevelopment of the VBN model in this study, resulting in the lack of comprehensiveness in the discussion on the roles of values and beliefs in promoting SEI. It is recommended for future research to consider exploring more beliefs-related antecedents with respect to the VBN model, such as biosphere values and egoistic values.

Secondly, the current study targeted only working adults in China. Furthermore, convenience sampling strategy was employed to gather respondents for the online questionnaire survey. The gathered sample varies across regions and populations, depending on the environment and resources. As a result, the discussed findings may not fully represent the views and perceptions on SEI of the entire Chinese population. Considering the depth and ongoing need for social enterprises in China as a developing country, it is recommended for future research to consider adopting the mixed-methods design, which combines both qualitative and quantitative methods, to explore this phenomenon.

## Conclusion

Social enterprises are generally more sustainable than the conventional philanthropic organizations, and the need for social enterprises in developing countries continues to be widespread and prevalent. Although certain conceptual and model debates still exist, a growing number of researchers and social groups attempt to map out relevant theories and practices. To date, there is still a lack of research on SEI. The purpose of this study was to discuss the motivational factors of social entrepreneurship. The proposed model in this study combined possible motivational factors with the VBN model. The testing of hypotheses was conducted based on a sample of Chinese working population to add support to the existing literature. In particular, this study empirically demonstrated the significant and positive influence of SFE, OTC, and SET on SET. Besides that, this study confirmed the significant and positive influence of SET, PRA, and OCE on PRN. This study also revealed the significant and positive influence of SET on PRA and the positive influence of PRA on OCE. Last but not least, this study proved the statistically significant and positive relationship of ISN, PRN, and SEI.

## Data availability statement

The original contributions presented in the study are included in the article/Supplementary material, further inquiries can be directed to the corresponding author.

## Ethics statement

Ethical review and approval was not required for the study on human participants in accordance with the local legislation and institutional requirements. The patients/participants provided their written informed consent to participate in this study.

## Author contributions

GJ, LS, and MM: conceptualization, investigation, methodology, writing-original draft preparation. QY and AM: conceptualization, methodology, formal analysis, and writing-review and editing. All authors contributed to the article and approved the submitted version.

## Conflict of interest

The authors declare that the research was conducted in the absence of any commercial or financial relationships that could be construed as a potential conflict of interest.

## Publisher’s note

All claims expressed in this article are solely those of the authors and do not necessarily represent those of their affiliated organizations, or those of the publisher, the editors and the reviewers. Any product that may be evaluated in this article, or claim that may be made by its manufacturer, is not guaranteed or endorsed by the publisher.

## Footnotes

^1^https://www.wjx.cn/

^2^https://webpower.psychstat.org/wiki/tools/index

## References

[ref1] Abutaleb S. El-Bassiouny N. M. Hamed S. (2021). A conceptualization of the role of religiosity in online collaborative consumption behavior. J. Islam. Mark. 12, 180–198. doi: 10.1108/jima-09-2019-0186

[ref2] Ajzen I. Fishbein M. (1970). The prediction of behavior from attitudinal and normative variables. J. Exp. Soc. Psychol. 6, 466–487. doi: 10.1016/0022-1031(70)90057-0

[ref3] Al-Jubari I. (2019). College students’ entrepreneurial intention: testing an integrated model of SDT and TPB. SAGE Open 9:215824401985346. doi: 10.1177/2158244019853467

[ref4] Avkiran N. K. Ringle C. M. (2018). Partial Least Squares Structural Equation Modeling: Recent Advances in Banking and Finance. Cham: Springer.

[ref5] Bacq S. Alt E. (2018). Feeling capable and valued: a prosocial perspective on the link between empathy and social entrepreneurial intentions. J. Bus. Ventur. 33, 333–350. doi: 10.1016/j.jbusvent.2018.01.004

[ref6] Bacq S. Janssen F. (2011). The multiple faces of social entrepreneurship: a review of definitional issues based on geographical and thematic criteria. Entrep. Reg. Dev. 23, 373–403. doi: 10.1080/08985626.2011.577242

[ref7] Barton M. Schaefer R. Canavati S. (2018). To be or not to be a social entrepreneur: motivational drivers amongst American business students. Entrep. Bus. Econ. Rev. 6, 9–35. doi: 10.15678/eber.2018.060101

[ref8] Bashir S. Khwaja M. G. Turi J. A. Toheed H. (2019). Extension of planned behavioral theory to consumer behaviors in green hotel. Heliyon 5:e02974. doi: 10.1016/j.heliyon.2019.e02974, PMID: 31872131 PMC6909088

[ref9] Bazan C. Gaultois H. Shaikh A. Gillespie K. Frederick S. Amjad A.. (2020). A systematic literature review of the influence of the university’s environment and support system on the precursors of social entrepreneurial intention of students. J. Innov. Entrep. 9:4. doi: 10.1186/s13731-020-0116-9

[ref10] Bertoldo R. Castro P. (2016). The outer influence inside us: exploring the relation between social and personal norms. Resour. Conserv. Recycl. 112, 45–53. doi: 10.1016/j.resconrec.2016.03.020

[ref11] Besika A. Schooler J. W. Verplanken B. Mrazek A. J. Ihm E. D. (2022). A relationship that makes life worth-living: levels of value orientation explain differences in meaning and life satisfaction. Heliyon 8:e08802. doi: 10.1016/j.heliyon.2022.e08802, PMID: 35146155 PMC8802095

[ref12] Blanton H. Köblitz A. McCaul K. D. (2008). Misperceptions about norm misperceptions: descriptive, injunctive, and affective ‘social norming’ efforts to change health behaviors. Soc. Personal. Psychol. Compass 2, 1379–1399. doi: 10.1111/j.1751-9004.2008.00107.x

[ref13] Bolino M. C. Grant A. M. (2016). The bright side of being prosocial at work, and the dark side, too: a review and agenda for research on other-oriented motives, behavior, and impact in organizations. Acad. Manag. Ann. 10, 599–670. doi: 10.5465/19416520.2016.1153260

[ref14] Bonan J. Cattaneo C. D’Adda G. Tavoni M. (2020). The interaction of descriptive and injunctive social norms in promoting energy conservation. Nat. Energy 5, 900–909. doi: 10.1038/s41560-020-00719-z

[ref15] Canlas I. P. Karpudewan M. Mohamed Ali Khan N. S. (2022). More than twenty years of value-belief-norm theory of environmentalism: what has been and yet to be done? Interdiscip. J. Environ. Sci. Educ. 18:e2269. doi: 10.21601/ijese/11801

[ref16] Cherrier H. Goswami P. Ray S. (2018). Social entrepreneurship: creating value in the context of institutional complexity. J. Bus. Res. 86, 245–258. doi: 10.1016/j.jbusres.2017.10.056

[ref17] Cho H. Chung S. Filippova A. (2015). Perceptions of social norms surrounding digital piracy: the effect of social projection and communication exposure on injunctive and descriptive social norms. Comput. Hum. Behav. 48, 506–515. doi: 10.1016/j.chb.2015.02.018

[ref18] Choi H. Jang J. Kandampully J. (2015). Application of the extended VBN theory to understand consumers’ decisions about green hotels. Int. J. Hosp. Manag. 51, 87–95. doi: 10.1016/j.ijhm.2015.08.004

[ref19] Cialdini R. B. (2003). Crafting normative messages to protect the environment. Curr. Dir. Psychol. Sci. 12, 105–109. doi: 10.1111/1467-8721.01242

[ref20] Cohen J. (2013). Statistical Power Analysis for the Behavioral Sciences. Cambridge, Massachusetts: Academic Press.

[ref1003] Crupi A. Liu S. Liu W. (2021). The top‐down pattern of social Innovation and social entrepreneurship. Bricolage and agility in response to Covid‐19: Cases from China. R and D Management 52, 313–330. doi: 10.1111/radm.12499

[ref21] De Bernardi P. Bertello A. Forliano C. Orlandi L. B. (2021). Beyond the “ivory tower”. Comparing academic and non-academic knowledge on social entrepreneurship. Int. Entrep. Manag. J. 18, 999–1032. doi: 10.1007/s11365-021-00783-1

[ref22] De Groot J. I. Bondy K. Schuitema G. (2021). Listen to others or yourself? The role of personal norms on the effectiveness of social norm interventions to change pro-environmental behavior. J. Environ. Psychol. 78:101688. doi: 10.1016/j.jenvp.2021.101688

[ref23] De Groot J. I. Steg L. (2009). Morality and prosocial behavior: the role of awareness, responsibility, and norms in the norm activation model. J. Soc. Psychol. 149, 425–449. doi: 10.3200/socp.149.4.425-449, PMID: 19702104

[ref24] Dijkstra T. K. Henseler J. (2015). Consistent partial least squares path modeling. MIS Q. 39, 297–316. doi: 10.25300/misq/2015/39.2.02

[ref25] Diochon M. (2013). Social entrepreneurship and effectiveness in poverty alleviation: a case study of a Canadian first nations community. J. Soc. Entrep. 4, 302–330. doi: 10.1080/19420676.2013.820779

[ref26] Dong S. Fioramonti D. Campbell A. Ebener D. (2017). Validation of the spiritual involvement and beliefs scale in a college student sample. J. Spiritual. Ment. Health 20, 167–184. doi: 10.1080/19349637.2017.1360169

[ref27] Doran R. Larsen S. (2015). The relative importance of social and personal norms in explaining intentions to choose eco-friendly travel options. Int. J. Tour. Res. 18, 159–166. doi: 10.1002/jtr.2042

[ref28] Dwivedi A. Weerawardena J. (2018). Conceptualizing and operationalizing the social entrepreneurship construct. J. Bus. Res. 86, 32–40. doi: 10.1016/j.jbusres.2018.01.053

[ref29] Ebbes P. Wedel M. Böckenholt U. Steerneman T. (2005). Solving and testing for regressor-error (in)dependence when no instrumental variables are available: with new evidence for the effect of education on income. Quant. Mark. Econ. 3, 365–392. doi: 10.1007/s11129-005-1177-6

[ref1002] Entrialgo M. Iglesias V. (2016). The moderating role of entrepreneurship education on the antecedents of entrepreneurial intention. Int. Entrepre. Manag. J. 12, 1209–1232. doi: 10.1007/s11365-016-0389-4

[ref30] Fayolle A. Liñán F. (2014). The future of research on entrepreneurial intentions. J. Bus. Res. 67, 663–666. doi: 10.1016/j.jbusres.2013.11.024

[ref1001] Fuller C. M. Simmering M. J. Atinc G. Atinc Y. Babin B. J. (2016). Common methods variance detection in business research. J. Bus. Res. 69, 3192–3198. doi: 10.1016/j.jbusres.2015.12.008

[ref31] Gärling T. (1999). Value priorities, social value orientations and cooperation in social dilemmas. Br. J. Soc. Psychol. 38, 397–408. doi: 10.1348/014466699164239

[ref32] George L. S. Park C. L. (2016). Meaning in life as comprehension, purpose, and mattering: toward integration and new research questions. Rev. Gen. Psychol. 20, 205–220. doi: 10.1037/gpr0000077

[ref33] Ghazali E. M. Nguyen B. Mutum D. S. Yap S. (2019). Pro-environmental behaviours and value-belief-norm theory: assessing unobserved heterogeneity of two ethnic groups. Sustainability 11:3237. doi: 10.3390/su11123237

[ref34] Gkargkavouzi A. Halkos G. Matsiori S. (2019). Environmental behavior in a private-sphere context: integrating theories of planned behavior and value belief norm, self-identity and habit. Resour. Conserv. Recycl. 148, 145–156. doi: 10.1016/j.resconrec.2019.01.039

[ref35] Golob U. Podnar K. Koklič M. K. Zabkar V. (2018). The importance of corporate social responsibility for responsible consumption: exploring moral motivations of consumers. Corp. Soc. Responsib. Environ. Manag. 26, 416–423. doi: 10.1002/csr.1693

[ref36] Hafenbrack A. C. Cameron L. D. Spreitzer G. M. Zhang C. Noval L. J. Shaffakat S. (2020). Helping people by being in the present: mindfulness increases prosocial behavior. Organ. Behav. Hum. Decis. Process. 159, 21–38. doi: 10.1016/j.obhdp.2019.08.005

[ref37] Hair J. F. Hult G. T. Ringle C. Sarstedt M. (2022). A Primer on Partial Least Squares Structural Equation Modeling (PLS-SEM), 3rd. Thousand Oaks, CA: SAGE Publications.

[ref38] Hair J. F. Hult G. T. Ringle C. M. Sarstedt M. Danks N. P. Ray S. (2021). Partial Least Squares Structural Equation Modeling (PLS-SEM) Using R: A Workbook. Berlin: Springer Nature.

[ref39] Heinicke F. König-Kersting C. Schmidt R. (2022). Injunctive vs. descriptive social norms and reference group dependence. J. Econ. Behav. Organ. 195, 199–218. doi: 10.1016/j.jebo.2022.01.008

[ref40] Hockerts K. (2017). Determinants of social entrepreneurial intentions. Entrep. Theory Pract. 41, 105–130. doi: 10.1111/etap.12171

[ref41] Hu X. Marlow S. Zimmermann A. Martin L. Frank R. (2019). Understanding opportunities in social entrepreneurship: a critical realist abstraction. Entrep. Theory Pract. 44, 1032–1056. doi: 10.1177/1042258719879633

[ref42] Hult G. T. Hair J. F. Proksch D. Sarstedt M. Pinkwart A. Ringle C. M. (2018). Addressing Endogeneity in international marketing applications of partial least squares structural equation modeling. J. Int. Mark. 26, 1–21. doi: 10.1509/jim.17.0151

[ref43] Jansson J. Dorrepaal E. (2015). Personal norms for dealing with climate change: results from a survey using moral foundations theory. Sustain. Dev. 23, 381–395. doi: 10.1002/sd.1598

[ref44] Joanes T. Gwozdz W. Klöckner C. A. (2020). Reducing personal clothing consumption: a cross-cultural validation of the comprehensive action determination model. J. Environ. Psychol. 71:101396. doi: 10.1016/j.jenvp.2020.101396

[ref45] Kaiser F. G. Hubner G. Bogner F. X. (2005). Contrasting the theory of planned behavior with the value-belief-norm model in explaining conservation behavior1. J. Appl. Soc. Psychol. 35, 2150–2170. doi: 10.1111/j.1559-1816.2005.tb02213.x

[ref46] Kautish P. Sharma R. (2019). Value orientation, green attitude and green behavioral intentions: an empirical investigation among young consumers. Young Consum. 20, 338–358. doi: 10.1108/yc-11-2018-0881

[ref47] Kim J. J. Hwang J. (2020). Merging the norm activation model and the theory of planned behavior in the context of drone food delivery services: does the level of product knowledge really matter? J. Hosp. Tour. Manag. 42, 1–11. doi: 10.1016/j.jhtm.2019.11.002

[ref48] Kim S. H. Seock Y. (2019). The roles of values and social norm on personal norms and pro-environmentally friendly apparel product purchasing behavior: the mediating role of personal norms. J. Retail. Consum. Serv. 51, 83–90. doi: 10.1016/j.jretconser.2019.05.023

[ref49] Kock N. (2015). Common method bias in PLS-SEM. Int. J. Ecollab. 11, 1–10. doi: 10.4018/ijec.2015100101

[ref50] Krueger N. F. Brazeal D. V. (1994). Entrepreneurial potential and potential entrepreneurs. Entrep. Theory Pract. 18, 91–104. doi: 10.1177/104225879401800307

[ref51] Lindeman M. Verkasalo M. (2005). Measuring values with the short Schwartz's value survey. J. Pers. Assess. 85, 170–178. doi: 10.1207/s15327752jpa8502_09, PMID: 16171417

[ref52] Makransky G. Borre-Gude S. Mayer R. E. (2019). Motivational and cognitive benefits of training in immersive virtual reality based on multiple assessments. J. Comput. Assist. Learn. 35, 691–707. doi: 10.1111/jcal.12375

[ref53] Morren M. Grinstein A. (2021). The cross-cultural challenges of integrating personal norms into the theory of planned behavior: a meta-analytic structural equation modeling (MASEM) approach. J. Environ. Psychol. 75:101593. doi: 10.1016/j.jenvp.2021.101593

[ref54] Nicholls A. (2010). The legitimacy of social entrepreneurship: reflexive isomorphism in a pre–paradigmatic field. Entrep. Theory Pract. 34, 611–633. doi: 10.1111/j.1540-6520.2010.00397.x

[ref55] Niemiec R. M. Champine V. Vaske J. J. Mertens A. (2020). Does the impact of norms vary by type of norm and type of conservation behavior? A meta-analysis. Soc. Nat. Resour. 33, 1024–1040. doi: 10.1080/08941920.2020.1729912

[ref56] Nordlund A. M. Garvill J. (2003). Effects of values, problem awareness, and personal norm on willingness to reduce personal car use. J. Environ. Psychol. 23, 339–347. doi: 10.1016/s0272-4944(03)00037-9

[ref57] Oh R. Fielding K. Nghiem L. Chang C. Carrasco L. Fuller R. (2021). Connection to nature is predicted by family values, social norms and personal experiences of nature. Glob. Ecol. Conserv. 28:e01632. doi: 10.1016/j.gecco.2021.e01632

[ref58] Parks-Leduc L. Feldman G. Bardi A. (2014). Personality traits and personal values. Personal. Soc. Psychol. Rev. 19, 3–29. doi: 10.1177/1088868314538548 24963077

[ref59] Rawhouser H. Cummings M. Newbert S. L. (2017). Social impact measurement: current approaches and future directions for social entrepreneurship research. Entrep. Theory Pract. 43, 82–115. doi: 10.1177/1042258717727718

[ref60] Rimal R. N. Yilma H. (2021). Descriptive, injunctive, and collective norms: an expansion of the theory of normative social behavior (TNSB). Health Commun. 37, 1573–1580. doi: 10.1080/10410236.2021.1902108, PMID: 33761815

[ref61] Ruiz-Rosa I. Gutiérrez-Taño D. García-Rodríguez F. J. (2020). Social entrepreneurial intention and the impact of COVID-19 pandemic: a structural model. Sustainability 12:6970. doi: 10.3390/su12176970

[ref62] Sarstedt M. Ringle C. M. Hair J. F. (2017). “Partial least squares structural equation modeling” in Handbook of Market Research. eds. Homburg C. Klarmann M. Vomberg A. (Cham: Springer)

[ref64] Schwartz S. H. (1977). Normative influences on altruism. Adv. Exp. Soc. Psychol. 10:5. doi: 10.1016/s0065-2601(08)60358-5

[ref65] Schwartz S. H. (1992). Universals in the content and structure of values: theoretical advances and empirical tests in 20 countries. Adv. Exp. Soc. Psychol. 25, 1–65. doi: 10.1016/s0065-2601(08)60281-6

[ref66] Schwartz S. H. Howard J. A. (1980). Explanations of the moderating effect of responsibility denial on the personal norm-behavior relationship. Soc. Psychol. Q. 43:441. doi: 10.2307/3033965

[ref67] Shenaar-Golan V. Walter O. (2020). Do emotional intelligence and self-compassion affect disordered eating perceptions? Am. J. Health Behav. 44, 384–391. doi: 10.5993/ajhb.44.4.2, PMID: 32553021

[ref68] Si H. Shi J. Tang D. Wu G. Lan J. (2020). Understanding intention and behavior toward sustainable usage of bike sharing by extending the theory of planned behavior. Resour. Conserv. Recycl. 152:104513. doi: 10.1016/j.resconrec.2019.104513

[ref69] Steg L. Lindenberg P. Keizer K. (2016). Intrinsic motivation, norms and environmental behaviour: the dynamics of overarching goals. Int. Rev. Environ. Resour. Econ. 9, 179–207. doi: 10.1561/101.00000077

[ref70] Steg L. Nordlund A. (2018). Theories to explain environmental behaviour. Steg L. Groot J.I.M. de, Environmental Psychology. New York: John Wiley and Sons Ltd., 217–227.

[ref71] Stern P. C. Dietz T. Abe T. Guagnano G. A. Kalof L. (1999). A value-belief-norm theory of support for social movements: the case of environmentalism. Hum. Ecol. Rev. 6, 81–97.

[ref72] Taso Y. Ho C. Chen R. (2020). The impact of problem awareness and biospheric values on the intention to use a smart meter. Energy Policy 147:111873. doi: 10.1016/j.enpol.2020.111873

[ref73] Tewari A. Mathur S. Srivastava S. Gangwar D. (2022). Examining the role of receptivity to green communication, altruism and openness to change on young consumers’ intention to purchase green apparel: a multi-analytical approach. J. Retail. Consum. Serv. 66:102938. doi: 10.1016/j.jretconser.2022.102938

[ref74] Thøgersen J. (2006). Norms for environmentally responsible behaviour: an extended taxonomy. J. Environ. Psychol. 26, 247–261. doi: 10.1016/j.jenvp.2006.09.004

[ref75] Tiwari P. Bhat A. K. Tikoria J. (2017). An empirical analysis of the factors affecting social entrepreneurial intentions. J. Glob. Entrep. Res. 7:9. doi: 10.1186/s40497-017-0067-1

[ref76] Tu B. Bhowmik R. Hasan M. K. Asheq A. A. Rahaman M. A. Chen X. (2021). Graduate students’ behavioral intention towards social entrepreneurship: role of social vision, innovativeness, social proactiveness, and risk taking. Sustainability 13:6386. doi: 10.3390/su13116386

[ref77] Ünal A. B. Steg L. Gorsira M. (2017). Values versus environmental knowledge as triggers of a process of activation of personal norms for eco-driving. Environ. Behav. 50, 1092–1118. doi: 10.1177/0013916517728991, PMID: 30473587 PMC6207993

[ref78] Ünal A. B. Steg L. Granskaya J. (2019). “To support or not to support, that is the question”. Testing the VBN theory in predicting support for car use reduction policies in Russia. Transp. Res. A Policy Pract. 119, 73–81. doi: 10.1016/j.tra.2018.10.042

[ref79] Wang C. Duan Z. Yu L. (2016). From nonprofit organization to social enterprise. Int. J. Contemp. Hosp. Manag. 28, 1287–1306. doi: 10.1108/ijchm-05-2014-0230

[ref80] Warnecke T. (2018). Social entrepreneurship in China: driving institutional change. J. Econ. Issues 52, 368–377. doi: 10.1080/00213624.2018.1469866

[ref81] Wong N. (2019). Injunctive and descriptive norms and theory of planned behavior: influencing intentions to use sunscreen. Womens Health Complications 2, 1–7.

[ref82] Zammitti A. Russo A. Santisi G. Magnano P. (2021). Personal values in relation to risk intelligence: evidence from a multi-mediation model. Behav. Sci. 11:109. doi: 10.3390/bs11080109, PMID: 34436099 PMC8389275

