# Supplementary material for: Social entrepreneurial intention among working adults: An emerging country context
Source: Front Psychol. 2023 Feb 13;14:1123198. doi: 10.3389/fpsyg.2023.1123198 (PMC9968742; doi:10.3389/fpsyg.2023.1123198)
Supplement: Supplementary file 1 [file Table_1.DOCX]

Survey Questionnaire

Decomposed Value-Belief-Norm Model to Predict Social Entrepreneurial Intention among Working Adults

Please indicate to what extent the following are important as a guiding principle in your life.

(Not very important [1] – Very important [7])

SEE1. Social power

SEE2. Authority

SEE3. Success

SEE4. Capability

SEE5. Ambition

OTC1. Gratification of desire

OTC2. Self-indulgence

OTC3. Daring

OTC4. Challenging life

OTC5. Creativity

OTC6. Independence

SFT1. World at peace

SFT2. Equality

SFT3. Wisdom

SFT4. Unity with nature

SFT5. Helpfulness

SFT6. Forgiveness

SFT7. Loyalty

SFT8. Broad mindedness, beauty of nature

SMP1. When I wrong someone, I make an effort to apologize.

SMP2. When I am ashamed of something I have done, I tell someone.

SMP3. I examine my actions to see if they reflect my values.

SMP4. I am thankful for all that has happened to me.

SMP5. I can find meaning in times of hardship.

SMP6. My life has a purpose.

Please indicate to what extent the following are agreeable to you. (Strongly Disagree [1] – Strongly Agree [7])

PRA1. Social inequality is a serious problem.

PRA2. Social problems need collective action to bring societal uplift.

PRA3. I am anxious about social challenges emerges from the social disparities.

PRA4. Social inequalities are the major reasons of economic challenges.

PRA5. Overall, I am concerned about social issues triggering the social problems.

OCE1. I think taking responsibility to solve social issues are important.

OCE2. It is important to offer employment opportunities to all segments of community.

OCE3. It is advisable to provide equal health and education opportunities for all.

OCE4. It is worthwhile to reduce the social differences between the communities.

OCE5. I feel responsible for the personal healthcare.

PSN1. I feel morally accountable to reduce the social disparity.

PSN2. People like me should use the work to curtail the social inequality.

PSN3. I feel morally obliged to make efforts to promote the social wellbeing of community.

PSN4. I morally think that working for community wellbeing is important, regardless of what others do.

PSN5. I feel personally obliged to work for the promotion of community wellbeing.

ISN1. Most of people in my companionship should work to reduce the social disparity of community.

ISN2. Most of significant people in my life are making efforts to bring social equality.

ISN3. Most of my peers willingly engage in promoting the social wellbeing although it might take more efforts.

ISN4. Most of my peers enthusiastically engage in social welfare of community even though it might be costly.

ISN5. Most of people among my peers should to work to achieve the community social wellbeing.

SEI1. I intend to start a social enterprise to promote social equalities.

SEI2. I will always try to make efforts to curtail the social inequalities in the future.

SEI3. I plan to use my knowledge and skills to uplift the community welfare.

SEI4. I would be willing to develop a social organization building prosper society.

SEI5. I can predict that I will engage myself in bridging the social differences in my community.
