# Supplementary material for: Social entrepreneurial intention among working adults: An emerging country context
Source: Front Psychol. 2023 Feb 13;14:1123198. doi: 10.3389/fpsyg.2023.1123198 (PMC9968742; doi:10.3389/fpsyg.2023.1123198)
Supplement: Supplementary file 2 [file Table_2.docx]

**Supplementary File 2.** Discriminant Validity

|  | SFE | OTC | SET | SMP | | PRA | | OCE | | PSN | | ISN | | | SEI | | |
| --- | --- | --- | --- | --- | --- | --- | --- | --- | --- | --- | --- | --- | --- | --- | --- | --- | --- |
| Fornell-Larcker Criterion | | | |  | |  | |  | |  | |  | | |  | | |
| SFE | 0.810 |  |  |  | |  | |  | |  | |  | | |  | | |
| OTC | 0.477 | 0.739 |  |  | |  | |  | |  | |  | | |  | | |
| SET | 0.486 | 0.475 | 0.827 |  | |  | |  | |  | |  | | |  | | |
| SMP | 0.490 | 0.483 | 0.612 | 0.803 | |  | |  | |  | |  | | |  | | |
| PRA | 0.480 | 0.462 | 0.486 | 0.535 | | 0.839 | |  | |  | |  | | |  | | |
| OCE | 0.463 | 0.415 | 0.596 | 0.593 | | 0.594 | | 0.843 | |  | |  | | |  | | |
| PSN | 0.468 | 0.427 | 0.505 | 0.586 | | 0.535 | | 0.581 | | 0.840 | |  | | |  | | |
| ISN | 0.439 | 0.438 | 0.473 | 0.567 | | 0.545 | | 0.535 | | 0.597 | | 0.861 | | |  | | |
| SEI | 0.436 | 0.457 | 0.399 | 0.499 | | 0.473 | | 0.460 | | 0.625 | | 0.620 | | | 0.846 | | |
| Heterotrait-Monotrait Ratio (HTMT_0.85_) | | | | | | | | | | | | | | | | |  |
| SFE |  |  |  |  |  | |  | |  | |  | | |  | | |  |
| OTC | 0.538 |  |  |  |  | |  | |  | |  | | |  | | |  |
| SET | 0.531 | 0.478 |  |  |  | |  | |  | |  | | |  | | |  |
| SMP | 0.552 | 0.518 | 0.669 |  |  | |  | |  | |  | | |  | | |  |
| PRA | 0.543 | 0.515 | 0.530 | 0.598 |  | |  | |  | |  | | |  | | |  |
| OCE | 0.513 | 0.435 | 0.650 | 0.662 | 0.660 | |  | |  | |  | | |  | | |  |
| PSN | 0.529 | 0.463 | 0.551 | 0.656 | 0.596 | | 0.646 | |  | |  | | |  | | |  |
| ISN | 0.492 | 0.480 | 0.512 | 0.628 | 0.602 | | 0.589 | | 0.660 | |  | | |  | | |  |
| SEI | 0.497 | 0.524 | 0.431 | 0.556 | 0.526 | | 0.507 | | 0.693 | | 0.682 | | |  | | |  |
| Loadings and Cross Loadings | | | | | | | | | | | | | | | |  |  |
| SFE1 | 0.794 | 0.395 | 0.329 | 0.366 | 0.396 | | 0.308 | | 0.380 | | 0.353 | | 0.381 | | |  |  |
| SFE2 | 0.802 | 0.367 | 0.342 | 0.346 | 0.348 | | 0.303 | | 0.364 | | 0.322 | | 0.368 | | |  |  |
| SFE3 | 0.874 | 0.409 | 0.438 | 0.444 | 0.425 | | 0.436 | | 0.413 | | 0.372 | | 0.361 | | |  |  |
| SFE4 | 0.811 | 0.359 | 0.489 | 0.451 | 0.387 | | 0.478 | | 0.376 | | 0.371 | | 0.294 | | |  |  |
| SFE5 | 0.762 | 0.408 | 0.339 | 0.355 | 0.384 | | 0.314 | | 0.358 | | 0.356 | | 0.380 | | |  |  |
| OTC1 | 0.346 | 0.691 | 0.270 | 0.288 | 0.377 | | 0.281 | | 0.265 | | 0.307 | | 0.307 | | |  |  |
| OTC2 | 0.207 | 0.583 | 0.078 | 0.140 | 0.222 | | 0.104 | | 0.172 | | 0.202 | | 0.296 | | |  |  |
| OTC3 | 0.291 | 0.713 | 0.245 | 0.305 | 0.263 | | 0.203 | | 0.240 | | 0.265 | | 0.340 | | |  |  |
| OTC4 | 0.381 | 0.828 | 0.352 | 0.404 | 0.349 | | 0.297 | | 0.367 | | 0.347 | | 0.396 | | |  |  |
| OTC5 | 0.359 | 0.822 | 0.410 | 0.392 | 0.366 | | 0.340 | | 0.353 | | 0.357 | | 0.350 | | |  |  |
| OTC6 | 0.449 | 0.771 | 0.539 | 0.470 | 0.420 | | 0.466 | | 0.401 | | 0.398 | | 0.346 | | |  |  |
| SFT1 | 0.423 | 0.397 | 0.809 | 0.515 | 0.399 | | 0.489 | | 0.409 | | 0.377 | | 0.321 | | |  |  |
| SFT2 | 0.397 | 0.396 | 0.852 | 0.513 | 0.402 | | 0.504 | | 0.430 | | 0.400 | | 0.319 | | |  |  |
| SFT3 | 0.384 | 0.385 | 0.836 | 0.487 | 0.419 | | 0.480 | | 0.416 | | 0.367 | | 0.311 | | |  |  |
| SFT4 | 0.424 | 0.413 | 0.826 | 0.517 | 0.413 | | 0.531 | | 0.425 | | 0.370 | | 0.303 | | |  |  |
| SFT5 | 0.410 | 0.409 | 0.825 | 0.536 | 0.416 | | 0.477 | | 0.419 | | 0.427 | | 0.365 | | |  |  |
| SFT6 | 0.408 | 0.397 | 0.834 | 0.494 | 0.405 | | 0.521 | | 0.413 | | 0.422 | | 0.349 | | |  |  |
| SFT7 | 0.372 | 0.373 | 0.808 | 0.494 | 0.369 | | 0.446 | | 0.414 | | 0.387 | | 0.373 | | |  |  |
| SFT8 | 0.391 | 0.367 | 0.823 | 0.486 | 0.390 | | 0.491 | | 0.411 | | 0.376 | | 0.295 | | |  |  |
| SMP1 | 0.406 | 0.337 | 0.543 | 0.787 | 0.418 | | 0.512 | | 0.443 | | 0.439 | | 0.329 | | |  |  |
| SMP2 | 0.374 | 0.388 | 0.403 | 0.753 | 0.401 | | 0.403 | | 0.442 | | 0.412 | | 0.400 | | |  |  |
| SMP3 | 0.413 | 0.402 | 0.539 | 0.837 | 0.456 | | 0.515 | | 0.489 | | 0.478 | | 0.386 | | |  |  |
| SMP4 | 0.363 | 0.371 | 0.476 | 0.828 | 0.430 | | 0.471 | | 0.501 | | 0.447 | | 0.399 | | |  |  |
| SMP5 | 0.412 | 0.408 | 0.505 | 0.829 | 0.439 | | 0.491 | | 0.484 | | 0.471 | | 0.460 | | |  |  |
| SMP6 | 0.393 | 0.425 | 0.475 | 0.783 | 0.434 | | 0.461 | | 0.464 | | 0.480 | | 0.435 | | |  |  |
| PRA1 | 0.377 | 0.325 | 0.381 | 0.418 | 0.817 | | 0.474 | | 0.412 | | 0.417 | | 0.347 | | |  |  |
| PRA2 | 0.414 | 0.391 | 0.466 | 0.481 | 0.842 | | 0.551 | | 0.469 | | 0.459 | | 0.381 | | |  |  |
| PRA3 | 0.407 | 0.396 | 0.375 | 0.432 | 0.844 | | 0.466 | | 0.455 | | 0.487 | | 0.412 | | |  |  |
| PRA4 | 0.421 | 0.401 | 0.391 | 0.449 | 0.851 | | 0.474 | | 0.441 | | 0.450 | | 0.408 | | |  |  |
| PRA5 | 0.393 | 0.423 | 0.419 | 0.460 | 0.841 | | 0.520 | | 0.463 | | 0.470 | | 0.436 | | |  |  |
| OCE1 | 0.418 | 0.376 | 0.487 | 0.509 | 0.524 | | 0.832 | | 0.517 | | 0.494 | | 0.423 | | |  |  |
| OCE2 | 0.380 | 0.347 | 0.505 | 0.493 | 0.478 | | 0.847 | | 0.485 | | 0.442 | | 0.391 | | |  |  |
| OCE3 | 0.353 | 0.318 | 0.509 | 0.490 | 0.482 | | 0.862 | | 0.471 | | 0.426 | | 0.362 | | |  |  |
| OCE4 | 0.400 | 0.360 | 0.495 | 0.512 | 0.513 | | 0.850 | | 0.494 | | 0.453 | | 0.399 | | |  |  |
| OCE5 | 0.399 | 0.343 | 0.514 | 0.493 | 0.502 | | 0.822 | | 0.477 | | 0.434 | | 0.361 | | |  |  |
| PSN1 | 0.434 | 0.373 | 0.473 | 0.505 | 0.492 | | 0.536 | | 0.821 | | 0.520 | | 0.505 | | |  |  |
| PSN2 | 0.339 | 0.330 | 0.340 | 0.431 | 0.422 | | 0.404 | | 0.811 | | 0.494 | | 0.558 | | |  |  |
| PSN3 | 0.406 | 0.347 | 0.434 | 0.503 | 0.426 | | 0.498 | | 0.844 | | 0.478 | | 0.495 | | |  |  |
| PSN4 | 0.392 | 0.366 | 0.442 | 0.506 | 0.449 | | 0.487 | | 0.868 | | 0.498 | | 0.525 | | |  |  |
| PSN5 | 0.392 | 0.378 | 0.429 | 0.516 | 0.456 | | 0.512 | | 0.856 | | 0.519 | | 0.544 | | |  |  |
| ISN1 | 0.404 | 0.404 | 0.423 | 0.491 | 0.479 | | 0.487 | | 0.527 | | 0.832 | | 0.528 | | |  |  |
| ISN2 | 0.360 | 0.384 | 0.407 | 0.469 | 0.479 | | 0.444 | | 0.485 | | 0.860 | | 0.513 | | |  |  |
| ISN3 | 0.371 | 0.361 | 0.403 | 0.483 | 0.454 | | 0.472 | | 0.516 | | 0.874 | | 0.550 | | |  |  |
| ISN4 | 0.386 | 0.356 | 0.402 | 0.501 | 0.461 | | 0.441 | | 0.510 | | 0.876 | | 0.546 | | |  |  |
| ISN5 | 0.368 | 0.381 | 0.402 | 0.492 | 0.472 | | 0.456 | | 0.531 | | 0.861 | | 0.531 | | |  |  |
| SEI1 | 0.339 | 0.360 | 0.238 | 0.347 | 0.363 | | 0.292 | | 0.438 | | 0.462 | | 0.798 | | |  |  |
| SEI2 | 0.398 | 0.412 | 0.343 | 0.422 | 0.427 | | 0.404 | | 0.550 | | 0.534 | | 0.863 | | |  |  |
| SEI3 | 0.380 | 0.386 | 0.382 | 0.447 | 0.412 | | 0.420 | | 0.551 | | 0.529 | | 0.858 | | |  |  |
| SEI4 | 0.351 | 0.366 | 0.353 | 0.445 | 0.379 | | 0.413 | | 0.524 | | 0.527 | | 0.859 | | |  |  |
| SEI5 | 0.372 | 0.406 | 0.356 | 0.442 | 0.416 | | 0.404 | | 0.566 | | 0.563 | | 0.850 | | |  |  |

**Note:** SFE: Self-Enhancement; OTC: Openness to Change; SET: Self-Transcendence; SMP: Sense of Meaning and Purpose; PRA: Problem Awareness; OCE: Outcome Efficacy; ISN: Injunctive Social Norms; PSN: Personal Norms; SEI: Social Entrepreneurial Intention;

**Source:** Author’s data analysis
